# The link between immunity and hypertension in the kidney and heart

**DOI:** 10.3389/fcvm.2023.1129384

**Published:** 2023-03-09

**Authors:** Lance N. Benson, Yunping Guo, Katherine Deck, Christoph Mora, Yunmeng Liu, Shengyu Mu

**Affiliations:** Department of Pharmacology and Toxicology, University of Arkansas for Medical Sciences, Little Rock, United States

**Keywords:** hypertension, immunity, inflammation, kidney, heart

## Abstract

Hypertension is the primary cause of cardiovascular disease, which is a leading killer worldwide. Despite the prevalence of this non-communicable disease, still between 90% and 95% of cases are of unknown or multivariate cause (“essential hypertension”). Current therapeutic options focus primarily on lowering blood pressure through decreasing peripheral resistance or reducing fluid volume, but fewer than half of hypertensive patients can reach blood pressure control. Hence, identifying unknown mechanisms causing essential hypertension and designing new treatment accordingly are critically needed for improving public health. In recent years, the immune system has been increasingly implicated in contributing to a plethora of cardiovascular diseases. Many studies have demonstrated the critical role of the immune system in the pathogenesis of hypertension, particularly through pro-inflammatory mechanisms within the kidney and heart, which, eventually, drive a myriad of renal and cardiovascular diseases. However, the precise mechanisms and potential therapeutic targets remain largely unknown. Therefore, identifying which immune players are contributing to local inflammation and characterizing pro-inflammatory molecules and mechanisms involved will provide promising new therapeutic targets that could lower blood pressure and prevent progression from hypertension into renal or cardiac dysfunction.

## Immunity, inflammation, and hypertension

Classically understood to activate as the “first line” in host defense without developing memory, innate immunity is comprised of dendritic cells, natural killer cells, neutrophils, macrophages, and other granulocytes (1). Although these cells typically function in infection clearance and homeostasis, a myriad of inflammatory responses have been identified when any of these immune cells become dysregulated such as in respiratory diseases (2) and aging (3). In the context of hypertension, monocytes, neutrophils, natural killer cells, and dendritic cells have been identified as present within the kidney and contributing to blood pressure elevation through several inflammatory mechanisms (4, 5). In contrast to innate immunity, adaptive immune cells (T and B lymphocytes) are classically understood to take longer for recruitment and activation through antigen-specific interactions, resulting in formation of memory and enabling more rapid and robust response following re-challenge with the infectious agent. Further sub-categories of T cells have been identified—each fulfilling a unique and critical biological niche, including CD4^+^, CD8^+^, and CD4^−^/CD8^−^ γδ T cells, within which further sub-classes have been identified (6). T cells, in particular, have been demonstrated to play a critical role in the development of hypertension (7), but B cell involvement cannot be ruled out (8). Certain cytokines, proteins that regulate the immune cellular function, have been directly linked to pro-inflammatory immune responses; however, the specific response can vary between target cell, surrounding tissue, timing, and cytokine concentration (9). In hypertension models, many cytokines characterized classically as “pro-inflammatory” have been found to arbitrate at least some of the animal model's blood pressure elevation or organ damage, including IFNγ (10–12), TNFα (13), RANTES (14), IL-1 (15, 16), IL-6 (17), IL-17 (12), IL-18 (18), and, potentially, IL-4 (18–20). IFNγ and IL-17, especially, have been confirmed to be elevated in clinical hypertension (21). We further demonstrated that IFNγ is critical for mediating CD8^+^ T cell infiltration and interaction within the kidney, driving increased sodium retention (10). Adoptive transfer of primary CD8^+^ T cells from hypertensive mice is able to induce salt sensitivity hypertension(SSH) in WT mice, however, adoptive transfer of primary CD8^+^ T cells from IFNγ KO hypertensive mice failed to induce SSHTN in WT mice (22). Collectively these data evidence the critical role of IFNγ in mediating immune-driven hypertension. For a thorough discussion of other cytokines within the kidney contributing to hypertension, we refer the reader to this review by Drs. Wen and Crowley (23).

Cardiovascular disease is the leading global killer, and hypertension is the primary contributing cause to this premature mortality (24). Of note, untreated or unsuccessfully managed hypertension has been demonstrated to result in organ dysfunction and damage—particularly within the heart and kidney (25). Either successful reduction of blood pressure or attenuation of hypertension induced organ dysfunction are key targets to prevent this premature mortality. Reducing blood pressure globally has proven challenging: not only due to resource scarcity -be that diet or medical- in certain areas (24) but also due to the clinical findings that the origin of elevated blood pressure is unknown in 90%–95% of cases (“essential hypertension”) (26) and up to 20%–30% of patient cases are resistant to current treatments (“resistant hypertension”) (27). Although patient compliance to drug regimens complicates treatment of this disease, true diagnosis of “resistant hypertension” requires assurance of adherence to administered therapeutics; as such, other confounding factors are clearly involved (28). Furthermore, the gut microbiota may further contribute to treatment resistance through direct enhancement of drug metabolism thereby reducing therapeutic effect (29, 30).

In light of recent studies, hypertension is presently understood as an inflammatory disease, involving immune cell invasion into organ tissue resulting in a myriad of cytokine expression changes, eventually driving endothelial dysfunction and organ damage (31–34). Through a combination of depletion and adoptive transfer studies, researchers were able to identify cells from both the innate and adaptive immunity that contributed to the development of hypertension (35) and subsequent tissue inflammation, especially the contributive roles of macrophages (36) and T cells (7). Other pre-clinical studies contributed to our current understanding that dendritic cells (37–40) and neutrophils (41, 42), are also participating in the pathogenesis of hypertension. Such pre-clinical studies have been corroborated clinically through the association of pro-inflammatory biomarkers with increased risk of treatment resistant hypertension in patients with chronic kidney disease (43), an independent association between several pro-inflammatory markers and higher blood pressure in healthy patients (44), and more than 50 years of observations of a myriad of immune cells accumulating in the blood vessels and kidneys of hypertensive patients (45). The key regulatory role of the kidney in the cardiovascular system has been well established (46); as such, identifying which immune cells are infiltrating the tissue and elucidating the signaling mechanisms involved are critical in improving treatment of this non-communicable disease. Indeed, recent advances have highlighted several new molecular targets involving the immune system that may serve as new targets for the treatment of resistant hypertension (10, 28, 47), but many pathways have not yet been elucidated. Additionally, unsuccessful management of hypertension has long been associated with progression to left ventricular hypertrophy and diastolic dysfunction within the heart (48–51), and the immune system has been implicated in this progression—particularly cardiac macrophages (52). Based on the critical functions of the kidney and heart in the cardiovascular system, this review will focus on the currently understood link between specific immune cells and hypertension through their interactions with -and within- the kidneys and heart, highlighting “key players” mediating these interactions.

## Inflammation and immunity: the bridge between renal dysfunction, hypertension, and kidney failure

**Figure 1:**
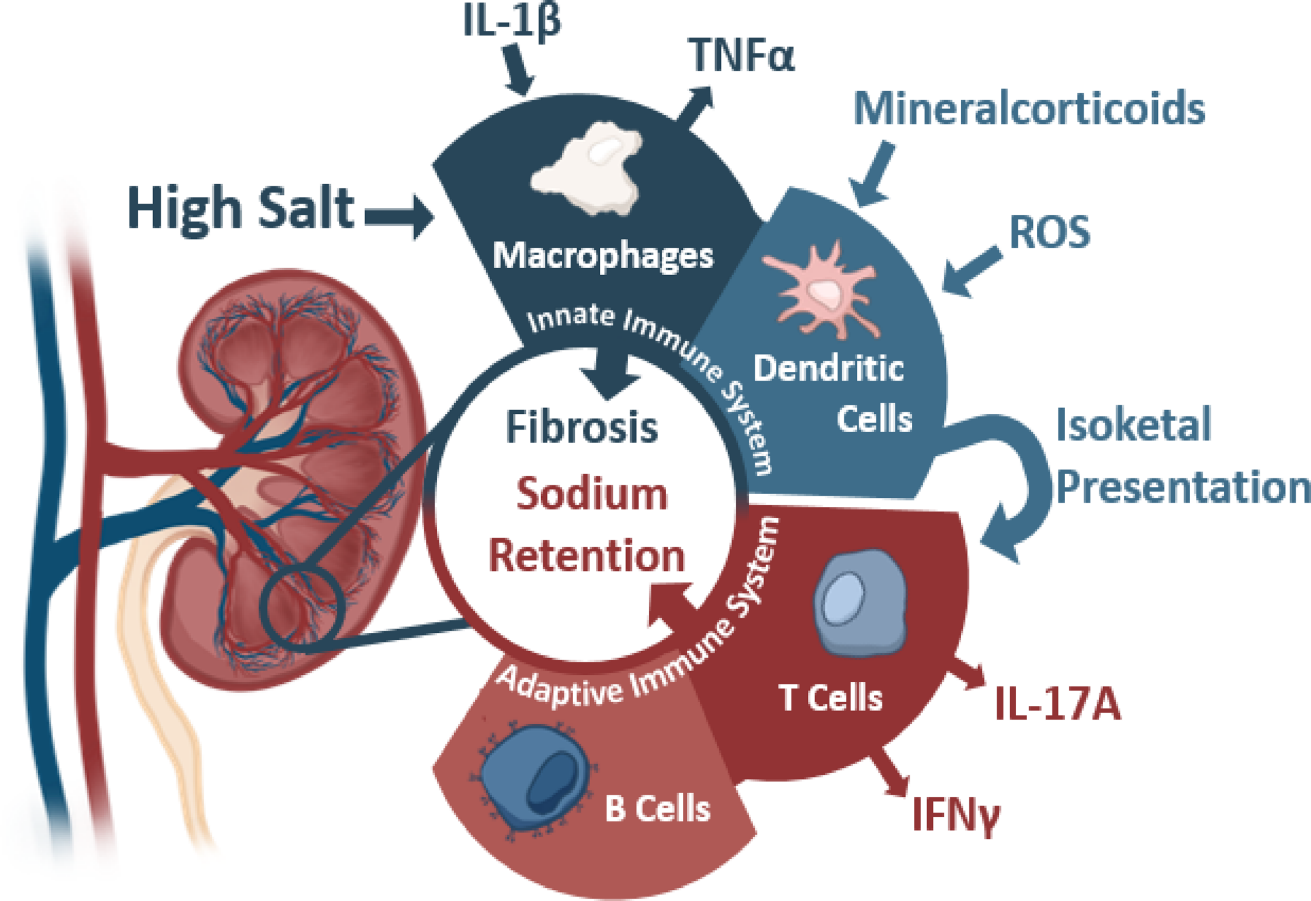
The contributions of the innate and adaptive immunity to kidney damage during hypertension with various activating stimuli and subsequent cytokine production. Sources below: Macrophages: IL-1β (Veiras et al., 2022, *Circulation Research,* secondary hypertension), TNFα (Singh et al., 2013 *American Journal of Physiology: Renal Physiology,* essential hypertension). Dendritic Cells: Mineralcorticoids (Araos et al., 2019 *Journal of Hypertension,* secondary hypertension), ROS (Lu et al., 2020 *Hypertension,* essential hypertension), and Isoketals (Kirabo et al., 2014, *Journal of Hypertension,* essential hypertension). T Cells: IL-17 (Kamat et al., 2015, *Hypertension,* essential hypertension), IFNγ (Benson et al., 2022 *Circulation Research* and Kamat et al., 2015, *Hypertension,* essential hypertension). B Cells: (Chan et al., 2015 *Hypertension*.)

Kidneys and blood pressure control have been intricately linked since Guyton's studies highlighted the importance of kidneys in pressure regulation through natriuresis (53). Defects in kidney function directly correlate to blood pressure dysregulation wherein transplantation of hypertensive kidneys into normotensive animals leads to the development of hypertension within that animal (54–59). Inversely, transplantation of a normotensive kidney into a previously hypertensive animal also can attenuate previously high blood pressure (57, 58). Mechanistically, several factors within the kidneys lead to high blood pressure through inappropriate sodium handling driving greater water retention. The generation of this pathology remains incompletely understood, but several mechanisms leading to this inappropriate handling have been hypothesized ranging from microvascular modeling to the interaction of various immune cells (Figure 1) (35, 59).

During the onset of hypertension, initial high blood pressure can cause permanent damage to the nephrons through arterial injury and glomerular ischemia, which progresses to nephrosclerosis. Within spontaneously hypertensive rats (SHR), renal vascular resistance increases within the afferent renal arterioles with arterial hypertrophy resulting in glomerular ischemia and reduced glomerular filtration (60, 61). Clinical evidence correlates these findings in several patient studies linking increased arteriole pressure with elevated hyaline deposits and arteriole wall thickness, indicative of renal damage (62, 63). Lower glomerular pressure, sensed *via* juxtaglomerular feedback, and lower luminal chloride, sensed *via* macula densa (64), promote increased renin production and activation of the renin-angiotensin-aldosterone system (RAAS). Increased secretion of renin leads through a series of hormones to increased angiotensin II, which acts as a vasoconstrictor and stimulates aldosterone secretion. Aldosterone will act on the distal nephron to increase sodium and water retention thereby increasing blood pressure (65). Damage from renal ischemia due to systemic hypertension can lead to secretion of various inflammatory cytokines, which, in turn, drives infiltration of immune cells and tubular atrophy.

Concurrently, the sympathetic nervous system (SNS) also plays a role in immune cell infiltration. Sensory neurons sense changes in blood pressure leading to the activation of the SNS, and chronic activation of SNS may lead to the development of hypertension as described in these reviews referenced here (65–67). One such experiment by Xiao et al., found that renal denervation blunted immune activation and blood pressure elevation in mice receiving angiotensin II (68), indicating some neural regulation of inflammation in hypertension. SNS activation also leads to increased release of renin, which elevates the blood pressure sensed by the kidney and is hypothesized to stimulate prostaglandin production (69). Catecholamines signal through the β2-adrenergic receptors on innate and adaptive immune cells which are involved in the development of cancer and autoimmune disorder. However, the clear pathway of how Catecholamines regulates immune cells is still unknown (65). Overall, overactivation of the SNS is often seen with hypertension, and its activity can lead to cytokine production and release (e.g., IFNγ and/or TNFα), which is associated with various tissue and organ damage in hypertensive patients resulting in subsequent damage as both the innate and adaptive immune systems accumulate and are key players in kidney damage (7, 35, 68, 70–75). An inverse relationship wherein inflammation influences nervous system function and signaling has been observed in viral infection (76), endometriosis (77), and arthritis (78). Considering the interplay between SNS activity and hypertension, it is possible that renal inflammation may, in turn, dysregulate SNS function in a feedback loop; however, further research is necessary to confirm if this relationship exists in hypertension.

Prostaglandins(PGs), which increase in production with salt loading, indirectly reduce sodium reabsorption and contribute to vasodilation beneficial for hypertensive individuals (65). In addition to their beneficial effects, PGs also increase vascular permeability and their chemotactic activity may attract leukocytes to the locally inflamed zone (79). Moreover, PGE2 has been found to stimulate IFNγ receptor expression on human lymphocytes (80), further implicating IFNγ signaling mechanisms in inflammation and hypertension. PGE2 has been shown to increase blood pressure and disrupt endothelial signaling in EP2/4 (E-prostanoid receptor) deficient mouse models (81–83) or in the presence of enhanced EP3 expression (84, 85), complicating the role of prostaglandins in hypertension.

## Innate immunity and the kidney in hypertension

Depletion of mononuclear phagocytes (MPs, includes macrophages and dendritic cells)) with liposome-encapsulated clodronate reduces fibrosis and glomerular sclerosis typical of salt-sensitive hypertension models (86). Activation of these cells can stem from a variety of effectors from the high salt environment to the activity of the SNS (68, 75, 87). High salt, specifically high sodium rather than high chloride or high osmolarity, triggers a pro-inflammatory state similar to the macrophage M1 state through p38 and ERK (87). Interestingly, this specific activation does not completely match the pro-inflammatory macrophage response seen with LPS stimulation. Macrophages activated by LPS eventually resolve towards an anti-inflammatory state whereas high salt-activated macrophages experienced suppression of this resolution (87). Activated MPS within hypertension leads to ROS production and generation of the cytokines IL-1β and TNFα (88), which is covered in greater detail in this review referenced here (89). Tubular IL-1β, in particular, has been demonstrated to recruit macrophages to the kidney leading to upregulation of ENaC and salt-sensitive hypertension in diabetic mice which are characterized by renal inflammation (16). By knocking out the IL-1R receptor in immune cells or knocking down IL-1β, the authors were able to prevent the switch to pro-inflammatory macrophages thereby preventing macrophage induced salt-sensitivity (16). Additionally, mineralocorticoid receptor may also participate in activated MPs-induced fibrosis during acute kidney injury (AKI), because mineralocorticoid receptor antagonist Finerenone providing protection against this development of renal fibrosis (90); SGK1, a commonly known downstream signaling molecule of ROS and mineralocorticoids, has also been linked to antigen presentation as a salt sensor in CD11c^+^ cells (expressed on dendritic cells or macrophages) (91) which leads to an increase in Epithelial sodium channel (ENaC) in these cells (92). Upregulation of ENaC in CD11c^+^ cells, in turn, correlated with increased activation, NADPH oxidase expression, and isolevuglandin-protein adducts (92).

Bridging the gap between innate and adaptive immunity, high salt not only promotes activation of MPs but also leads to the formation of isolevuglandins, also referred to as isoketals (37, 92). Isolevuglandins are a product of arachidonic acid generated as the result of oxidative stress, which is both a product of -and contributor to- hypertension and renal dysfunction (93). Harrison and colleagues identified the accumulation of isolevuglandins during hypertension and the development of these into neoantigens by dendritic cells. Hence, these dendritic cells have the capability of presenting isolevuglandins to T cells leading to their activation, which plays an important role in the changes within the kidney during hypertension (37).

## Adaptive immunity and the kidney in hypertension

In his landmark study, Guzik et al., demonstrated adoptive transfer of T cells restored the hypertensive response to angiotensin II in RAG1^−/–^ (immunodeficient) mice (7). Many other studies confirmed the critical role of the T cell in the development of hypertension, including alternative animal models (94). De Miguel et al., for example, found that T cells invaded into the kidney of Dahl salt-sensitive rats on high salt diet, and administration of the immunosuppressant tacrolimus blunted T cell invasion within the kidney, reduced renal damage, and lowered blood pressure (95). Subsequent studies were conducted to identify the specific subtypes of contributing T cells, resulting in the identification of a variety of T cells that become active within hypertension including γδ, CD4^+^, and CD8^+^ T cells (5). γδ T cells hold the most similarities with the innate immune system and are capable of antigen presentation. Within hypertension, these γδ T cells as well as CD4^+^ T cells function by aiding in the activation of CD8^+^ T cells or through cytokine production, including IFNγ and IL-17A (5). In particular, γδ T cell deficient mice showed blunted hypertensive response and endothelial dysfunction to angiotensin II, and increased numbers of these T cells have been found in the spleens of hypertensive mice (96).

CD8^+^ T cells not only contribute to cytokine production in general inflammatory responses but we also found they interact directly with the nephron to increase sodium retention by stimulating an increase in the sodium chloride cotransporter (NCC) in the DOCA + salt model of hypertension (97). Additionally, splenic CD8Ts from hypertensive mice can generate salt sensitive hypertension in recipients (97). We further found the cytokine IFNγ was critical for this infiltration to take place in the DOCA + Salt model (10) and adoptive transfer models of hypertension (22). Of note, IFNγ from CD8^+^Ts stimulates the IFNγ receptor expressed within the distal convoluted tubule to promote interaction between the CD8^+^T and tubule (10). Additionally, Carnevale et al., found CD8Ts not only increased resistance in arteries in a three-dimensional culture model of hypertension but also exhibit enhanced gene pathway upregulation to chemotaxis and response to IFNγ in pre-hypertensive mice (98). The crucial role of IFNγ—along with IL-17—in immune-cell mediated hypertension was previously characterized by Kamat et al., in the angiotensin II model of hypertension (12). Clinically, hypertensive human CD8^+^T IFNγ production and CD4^+^T IL-17 has been found to be significantly elevated (21). Youn et al., also found pro-inflammatory immunosenescent CD8^+^Ts (expressing higher perforin, granzyme B, IFNγ, and TNFα) are increased in peripheral blood of hypertensive patients (99).

Within CD8^+^ T cells a variety of memory populations are generated upon activation including an effector memory T cell population that can enter and exit from lymphoid tissue to non-lymphoid tissue. Once activated, these T cells remain primed to the stimulus and become easier to activate. In a variety of models, several studies have found a dendritic cell-mediated increase in this effector memory population during hypertension (100–102). Trott et al., confirmed that an oligoclonal population of CD8^+^Ts can be found in the kidney of angiotensin II-induced hypertensive mice (103); however, the subtypes of CD8^+^Ts present have not yet been characterized. The development of these populations lead to the potential for continual T cell activation and the exertion of their effects on the kidney. Accordingly, outside of the physical damage due to the high pressure within the kidney, CD8^+^ T cells may have a long-lasting ability to become activated and lead directly to the inappropriate sodium handling within the nephron that drives water retention.

Regulatory T cells (T_regs_)—CD4+ T cells that express CD4, CD25, and FOXP3—are classically understood to reduce inflammation and play an anti-proliferative role in immune responses (104); as such, these cells may play a protective role in the kidney during the development of hypertension. Expansion of T regulatory cells through low-dose IL-2 administration has been shown to lower blood pressure induced *via* systemic lupus erythematosus in mice; as such, these cells may play a protective role in hypertension (105). Indeed, increased numbers of T_regs_ in female rats may explain the reduced DOCA + Salt induced hypertension in female rats compared to males, and depletion of *T*_regs_ increased female but not male rat blood pressure elevation and renal fibrosis to DOCA (106). Such a protective role may involve an immunosuppression independent mechanism; as such, further research is needed to clarify the protective, antihypertensive role of regulatory T cells in the kidney (107).

In addition to T cells, B cells have been observed to increase within- and invade- the hypertensive kidneys. B cells alone may not lead to the development of hypertension, as adoptive transfer of T cells and not B cells from hypertensive mice into normotensive Rag1^−/–^ mice (which lack B and T cells) resulted in hypertension (7). However, in mice that lack mature B cells through B-cell activating factor receptor deficiency, hypertension was attenuated in the Ang II model (8). B cells play a role in the accumulation of MPS and T cells, which may lead to the kidney pathologies observed during hypertension.

## Inflammation and immunity: the bridge between hypertension and congestive heart failure

Chronic hypertension has been identified as the leading cause of congestive heart failure (108). The progression of hypertension into cardiac dysfunction, and that of cardiac dysfunction into heart failure, is a multi-faceted progression which often involves morphological and molecular biological changes in the cardiomyocytes, alterations within the surrounding microenvironment, and physical changes to the heart. In addition to playing a role in the perturbation of blood pressure, often through salt-sensitive inappropriate sodium handling (109), decades of research have shown that immune cells are partially culprit in the onset of hypertension, the progression of cardiac dysfunction to heart failure and, possibly, even the progression of hypertension to cardiac dysfunction (41).

Either hypervolemia or increased peripheral resistance increase the pumping force requirements of the heart, especially within the left ventricle (110), to overcome the pressure in the aorta to successfully deliver blood to the rest of the body. This increase in pressure within the ventricle causes excessive stretching, resulting in cardiomyocyte injury and subsequent cardiac hypertrophy—a process mediated by the immune system (111). Furthermore, immune regulation of T cells, macrophages, fibroblasts, and dendritic cells all play a role in the worsening of cardiac function in hypertension and heart failure; as such, immunological targets appear to be promising targets to increase heart health and function in treatment of heart disease (112).

Collagen deposition in cardiac fibrosis plays an important role in the maintenance of the heart. Fibrosis functions in both maintaining homeostasis—especially within the interstitium—and in repairing physical injury that occurs. Fibrotic remodeling is a complex function that involves the secretion and deposition of connective molecules, most importantly Collagens I and III, into the interstitial and perivascular spaces. However, excessive deposition of these connective molecules can impair cardiac function. The increased deposition of molecules within the extracellular matrix (ECM) stiffens the heart chambers, thereby inhibiting the heart from expanding and contracting efficiently, and, ultimately, resulting in increased cardiac stress to maintain homeostatic output. As this fibrotic deposition progresses, cardiac output becomes sufficiently reduced to cause damage to other organs and damage to cardiac cells due to impaired circulation and increased pressure, a pathology so common that 50% of heart failure patients present with this increased cardiac load due to fibrosis (52, 113). Therefore, preventing fibrosis in heart failure remains an attractive target to help preserve cardiac function.

Matrix Metalloproteinases (MMPs) are a family of proteins that help regulate ECM deposition and degradation, many of which are produced by macrophages and other immune cells (114). In particular, MMP-9 has been implicated in a wide range of functions, including the degradation of ECM (115). During cardiac dysfunction, when the fibrotic response is excessive, MMP9 is produced to chaperone the correct degradation and disposition of molecules for proper ECM development and scar formation. However, it has been noted that the expression of MMP9 is reduced in patients with congestive heart failure (116), potentially indicating an impaired ability to degrade excess collagen and further highlighting the importance of extracellular matrix homeostasis.

## Innate immunity and cardiac fibrosis

Macrophages, originating from monocytes found in the circulation, express a variety of markers and serve a great deal of roles in innate immunity as well as mediate adaptive immune response by cross-talking with B and T cells (117). These cells are recruited to injured or infected cells *via* chemokine signaling (e.g., CCL2,CXCL1, CX3CL1) from a number of other cells including fibroblasts, endothelial cells, and epithelial cells (118). Of note, cardiac-resident macrophages appear to play a more protective role against fibrosis than monocyte-derived macrophages that infiltrate the heart and promote hypertrophy, but further research is needed to clarify the distinction in responses between monocyte derived macrophages and cardiac resident (119). There are traditionally understood to be two main macrophage phenotypes: M1 and M2 (120).

M1 macrophages are considered “traditionally activated” and become active upon exposure to IFN-γ and TLR stimulation (120). These macrophages are considered pro-inflammatory and secrete inflammatory cytokines such as IL-1β, TNF, and IL-12 (121). In a neonatal injured heart model, inhibition of cyclooxygenase-2 resulted in increased M1 macrophage recruitment at the wound site which may contribute to the treatment induced suppressed cardiac hypertrophy and fibrosis (122); however, resolution-phase macrophages can exhibit M1 markers yet express a unique inflammatory phenotype (123), making the role of M1 macrophages in cardiac fibrosis unclear. Depletion of macrophages (both phenotypes) has been shown to reduce cardiac remodeling in the Dahl salt-sensitive rat (124), but did not identify the key contributing phenotype.

In contrast to M1 macrophages, M2 macrophages are anti-inflammatory and pro-fibrotic in function; as such, the M2 phenotype may especially contribute to the fibrogenesis in hypertensive hearts.

M2 macrophages are “alternatively activated”(activated by IL-13, CSF-1, IL-4, IL-10, and TGF-β) (121, 125, 126). These cytokines, commonly produced by Th2 T cells, have been shown to be directly linked to the production of collagens and the onset of fibrosis (126), potentially through their stimulation of M2 macrophages. Furthermore Angiotensin II and cations have been implicated in the transition of fibroblasts to myofibroblasts capable of collagen production and deposition (127). The M2 macrophage, alone, is not capable of depositing the collagen seen in fibrosis; rather, these cells are able to promote the transition of the homeostatic fibroblasts into fibrosis-inducing myofibroblasts (128). Macrophages, once activated at the site of injury, can recruit more macrophages and circulating fibroblasts, leading to increased inflammation and fibrosis (127).

M2 macrophages induce myofibroblast differentiation through a number of mechanisms such as the production of TGF-1β. A recent study by Murray et al., implicated IL-13, TGF-β1, and CCL2 axis in the hyper-stimulation of myofibroblasts in a model of idiopathic pulmonary fibrosis with a usual interstitial pneumonia pathology (IPF/UIP) (129). IL-13, TGF-β1, and CCl2 together appeared to result in a synergistic effect in the activation of myofibroblasts. Though these results have not been explored in the heart specifically, they indicate the M2 macrophage can exacerbate organ fibrosis, which may be relevant in hypertension and heart failure through the same myofibroblastic axis.

### TGF-β

TGF-β plays a critical role in the onset and progression of fibrosis through myofibroblast differentiation (130, 131). TGF-β signals through the SMAD pathway inside of the homeostatic fibroblasts and induces the assembly of SMADs 2 and 3 into SMAD 4. SMAD 4 and its R-Smad counterparts then form a complex in the nucleus and modify the gene expression of the fibroblast into a myofibroblast (132). As such, TGF-β and its downstream effects are potential targets for the ablation of fibrosis in hypertension and heart failure. When translated, TGF-β is associated with its negative regulator, the latency associated protein (LAP) (133). TGF-β function can be arbitrated through multiple mechanisms, including through interaction with αVβ6, an inflammation-associated integrin expressed on epithelial cells (134). Munger et al., demonstrated that, though there are multiple methods for TGF-β to be activated, αVβ6 interaction is sufficient to initiate TGF-β signaling (135–138).

### IL-10 and CCL2

IL-10 is an anti-inflammatory cytokine produce by a myriad of cells including macrophages, CD4^+^ and CD8^+^ T cells, B cells, other monocytic cells (139). IL-10 targets M2 macrophages and activates them to a collagen-producing, pro-fibrotic phenotype (140). Pro-fibrotic macrophages play a critical role in the pathological progression of hypertension to diastolic dysfunction and heart failure (52). Furthermore, it has been shown that IL-10 deletion in a murine heart failure model attenuates erroneous fibrotic response and reduces mortality (52), suggesting IL-10 signaling cascades may be relevant therapeutic targets to prevent the progression of hypertension into heart failure.

**Figure 2:**
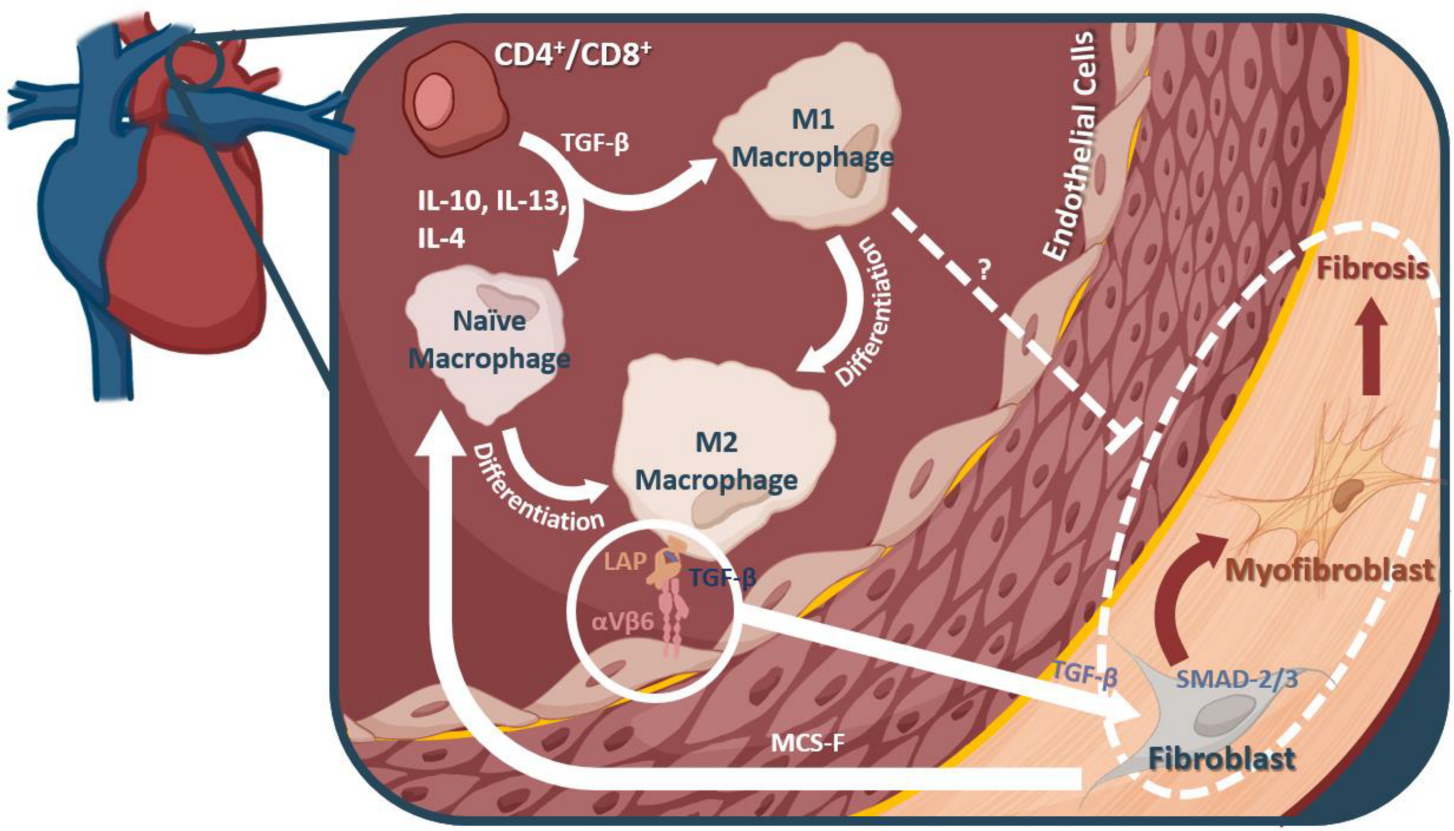
Macrophages and their mechanistic role in fibrosis. M2 macrophages can interact with αVβ6, thereby releasing a TGF-β inhibitor protein, and allowing them to interact with fibroblasts in the heart. TGF-β leads to myofibroblast differentiation from fibroblasts *via* the SMAD2/3 pathway. It is these myofibroblasts that are thought to account for much of the fibrotic remodeling in many diseases, including diastolic dysfunction and heart failure.

Macrophages, their differentiation, and their signaling molecules present potentially valuable targets for preventing the onset of fibrosis during hypertension and heart failure (outlined in Figure 2). However, the progression of fibrosis in hypertension and heart failure is not yet fully understood; as such, other pro-inflammatory immune cells may be contributing to the pathology. In a pressure overload mouse model of heart failure through transverse aortic constriction, Wang et al., found that depletion of CD11C^+^ cells blunted left ventricular fibrosis and hypertrophy, implicating dendritic cells in contributing to this pro-inflammatory state (141), but CD11C^+^ macrophages may be relevant to this model (142). In the DOCA + Salt model of hypertension, depletion of CD11C^+^ cells attenuated progression into cardiac hypertrophy and fibrosis, further highlighting the role of dendritic cells (143). Natural killer (NK) cells, on the other hand, may play a protective role *via* attenuating cardiac fibrosis (144). Additional research is needed to clarify the role of other innate immune cells in hypertension induced cardiac fibrosis.

## Adaptive immunity and cardiac fibrosis

### CD4^+^T cells

T-helper cells—a subset of CD4^+^ T cells—are further subdivided into Th1, Th17, Th2, Th3, and Tr1. These cells serve a variety of functions in response to both foreign antigens and injury. Decades of literature, especially recent studies, have identified CD4^+^T cells in the development and progression of cardiomyopathy (145). Laroumanie et al., recently implicated CD4^+^T cells in the progression of hypotrophy to heart failure in a myocardial infarction murine model (109). Utilizing multiple mice strains including, RAG2-KO, CD8^+^-KO, and MHCII-KO, the authors found that CD4^+^T cells appear to be the primary drivers of cardiac fibrosis after TAC-induced heart failure. Furthermore, adoptive transfer of T cells from CD8^+^-KO mice (only CD4^+^T cells transferred) to RAG2-KO (immune incompetent) mice resulted in an increase of fibrosis in the recipients (109).

In the same study, to determine a mechanism by which CD4^+^T cells increase fibrosis, Lysyl oxidase (LOX), an amine oxidase that influences cross-linking between elastin and collagen, expression was investigated LOX is known to play a role in ECM development and fibrotic disease (146, 147). The researchers found no significant difference between pro-LOX production between WT and RAGII-KO mice; however, they showed that the ratio of mature LOX to pro-LOX increased in WT mice—hinting at some interaction from the CD4^+^T cells that might induce the maturation of LOX in heart failure thereby driving elastin and collagen cross-linking. While preventing the aberrant increase in LOX maturation may prevent the progression of hypotrophy to heart failure, the production of LOX from pro-LOX is not fully understood and may be dependent on a number of factors, complicating the development of therapies (109).

If pro-inflammatory T cells are contributing to the development of hypertension and subsequent cardiac fibrosis, it would likely follow that regulatory T cells may play a protective effect. Indeed, Wang et al., found that increased *T*_reg_ presence (induced by IL2/JES6-1 treatment) blunted left ventricular hypertrophy in the transverse aortic constriction model of heart failure (148); as such, Tregs may play a protective role in hypertension induced cardiac fibrosis, but further research is needed to outline the protective mechanisms involved.

### CD8^+^T cells

The extent of the role of either CD4^+^ or CD8^+^ T cells in the progression of hypertension and cardiovascular disease is not yet clear. As previously mentioned, Laroumanie et al., provided evidence pointing toward the role of CD4^+^ T cells in the progression of heart failure (109); however, in tandem with their CD4^+^ counterparts, other evidence supports that CD8^+^ T cells may also play a critical role in cardiovascular disease. In their 2019 study on immune cell activity post myocardial infarction, IIatovskaya et al., examined CD8^+^ T cell-specific effects by comparing the progression of scar formation after TAC in WT or CD8^+^-deficient tm1Mak mice. When CD8^+^ T cells were ablated, TAC mice exhibited abnormal scar formation (149). Additionally, they found a significant increase of mature LOX when CD8^+^ T cells were present, in contrast to the results found in the study by Laroumanie et al. (109) Regardless of immune cell source, it is evident that LOX production plays some causal role in the development of cardiac-straining scar tissue (109, 149).

**Figure 3:**
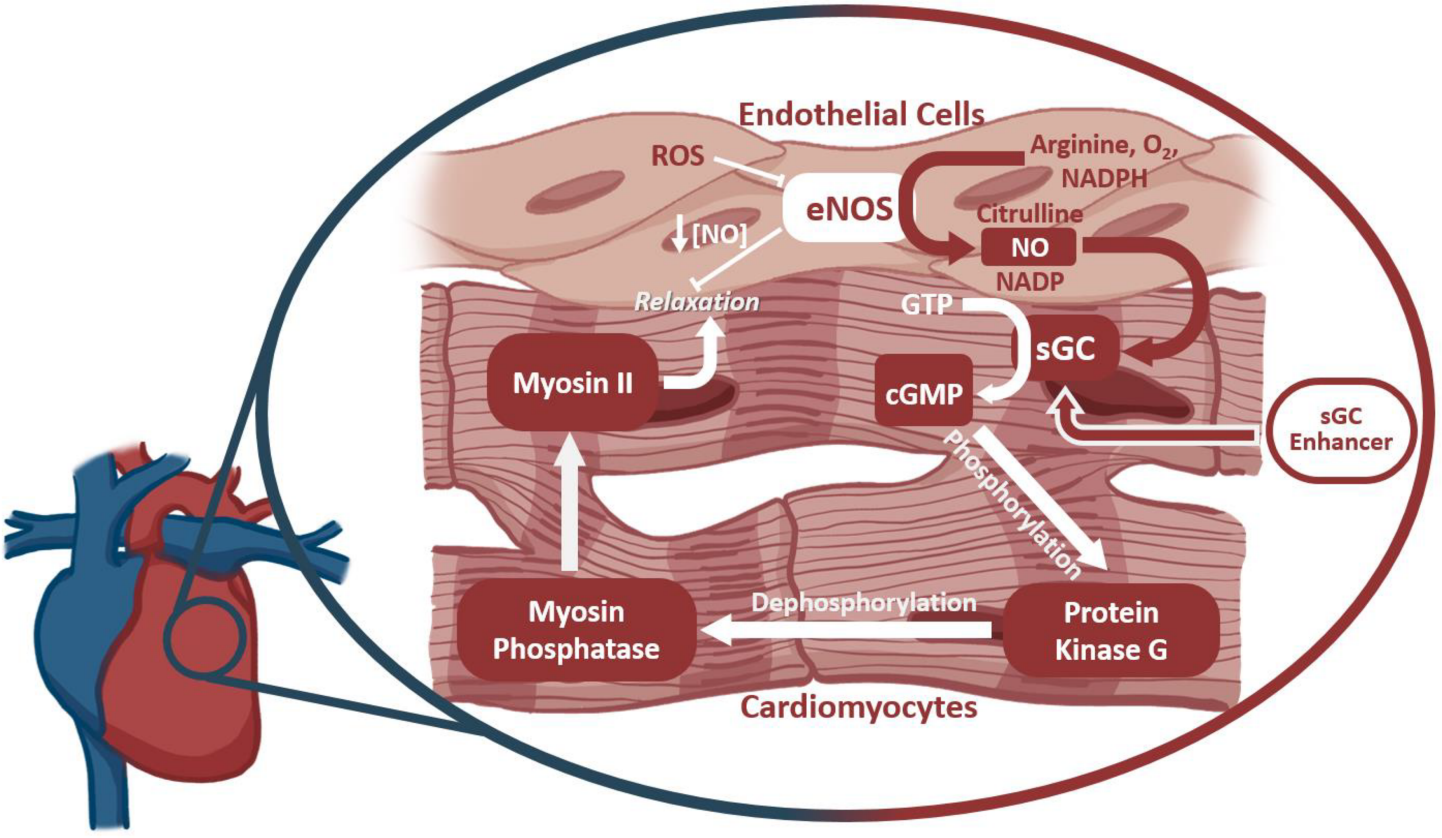
The outcome of ROS production on cardiomyocyte function. Soluble Guanylate Cyclase (sGC) drives Myosin II dephosphorylation and smooth muscle relaxation *via* production of the secondary messenger cGMP. sGC is activated by intercellular NO produced by endothelial nitric oxide synthase (eNOS). Reactive oxygen species inhibit eNOS activity, thereby affecting cardiomyocyte function further downstream. sGC enhancers target sGC at an allosteric site to increase its sensitivity to NO, thereby rescuing function despite lower NO availability.

Reactive oxygen species (ROS) are a hallmark of many diseases, including cancer, heart disease, and other organ diseases (150). Several potential mechanisms are available for a T cell to induce ROS release or production, such as the rupture of phagocytes that can sequester ROS (151). Excessive ROS production due to T cell activity can negatively affect contractility within the heart, among other dysfunctions. One such mechanism, as depicted in Figure 3, has been identified to involve Myosin II, which relies on myosin phosphatase to be dephosphorylated, leading to the relaxation of the muscle fibers. Myosin phosphatase is activated by protein kinase G, which, previously, had been activated by secondary messenger cGMP. This cGMP is produced by soluble Guanylate Cyclase (sGC) in the heart. sGC is activated by endogenous NO, which is produced by eNOS. eNOS is known to be inhibited by increased levels of intracellular ROS; as such, downstream contractility regulated by eNOS will be negatively impacted by increased ROS (152). By this mechanism, T cell activity in the failing heart might indirectly lead to decreased cardiomyocyte relaxation, which could further exacerbate cardiac stress and the progression to heart failure. Therapies for eNOS inactivity exist in which sGC is stimulated by small molecules to be more reactive to decreased levels of NO (153). Further research is needed to determine whether the ablation of T cells might have a protective role on this function of the cardiomyocytes through reducing inflammatory ROS production (153, 154).

## Current antihypertensive therapeutic treatments effect on immune function and inflammation

**Figure 4:**
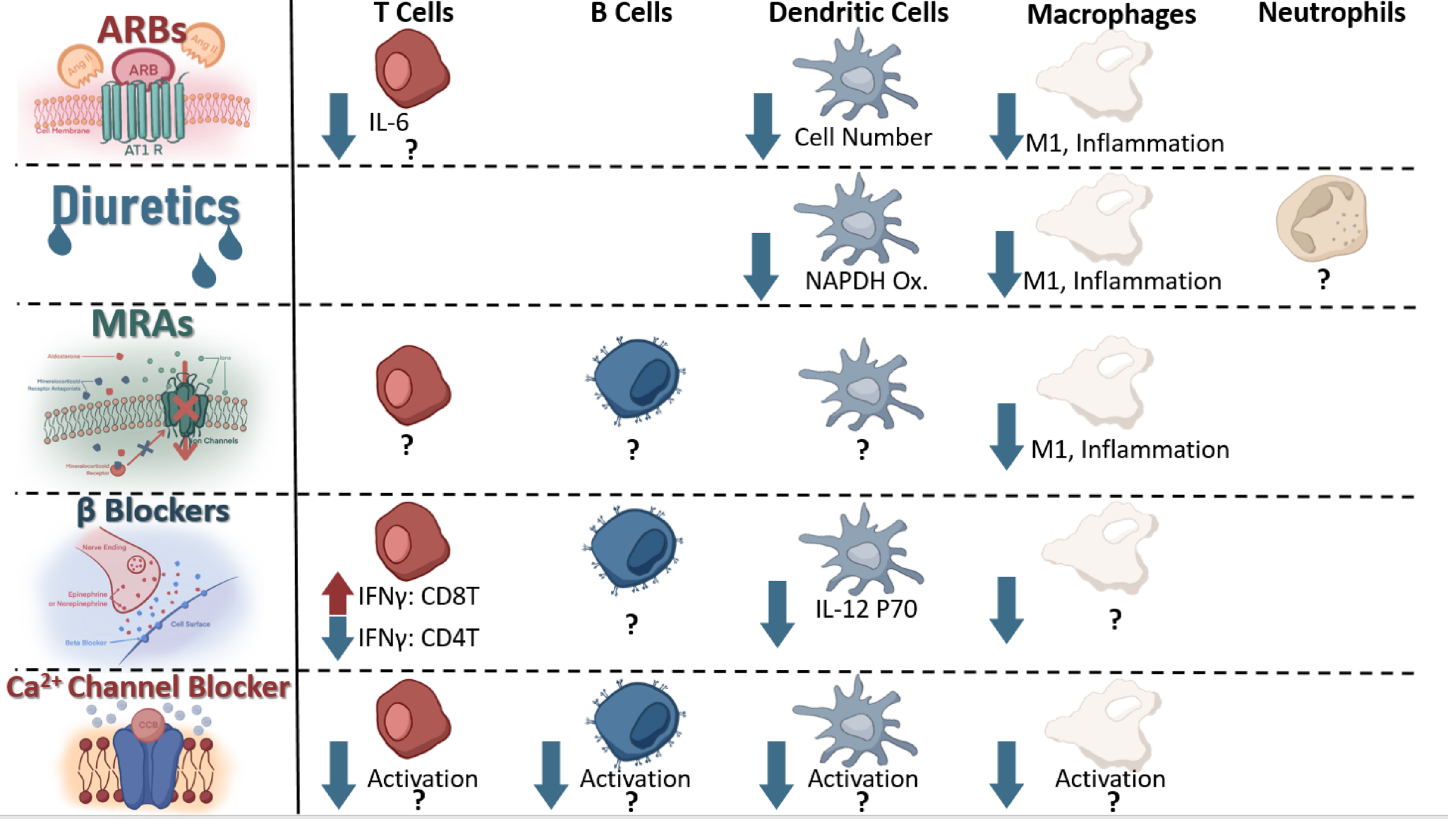
Immune cells known to express receptors antagonized by current antihypertensives and known change in signaling or function as a result of receptor antagonism.

Systemic administration of any drug will be expected to result in eventual off-target effects (155); as such, it should be unsurprising to consider that systemic administration of antihypertensives may result in alterations of off-target organ -or cellular- functions. Although such unintended molecule-receptor interactions are typically referred to as toxicities, it is also possible that the desired phenotype (i.e., lowering blood pressure) may be achieved through some of these very same “unintended” effects. To this end, it can become relevant to consider if receptors targeted by antihypertensives are also expressed and functional on other cells—such as immune cells that play a critical role in the development of hypertension (31, 35, 156). An excellent discussion by Felkle et al., regarding the role of ACEi, ARBs, CCBs, β-blockers, and thiazides in immune function modulation can be found referenced here (157). For the purpose of this review, we will provide a brief outline of several receptors and current understanding of expressing immune cell types and potential contributing mechanisms (Figure 4).

### Angiotensin II type 1 receptor blocker

The AT1 receptor, target of ARBs such as Candesartan, was described on splenocytes in 1999 (158), and further experiments have found this receptor on T cells (159), macrophages (160), and dendritic cells (161). Using an [3H] thymidine incorporation assay, Nataraj et al., found that increased Angiotensin II concentration stimulated proliferation of splenocytes, and inhibition of the AT1 receptor using Losartan resulted in reduced AngII binding to splenocytes (158). This link between angiotensin II and splenocyte proliferation indicated a potential for AT1 receptor antagonism to repress immune function. Indeed, Nataraj et al., found that AT1A-receptor deficient mouse graft recipients showed increased allograft survival when coupled with subtherapeutic doses of the calcineurin inhibitor cyclosporine, which showed no significant effect on AT1A receptor proficient mice, indicating a potential AT1-calcinuerin regulation pathway within immune cells (158). Such regulation opens the possibility of ARBs to influence immune function in addition to reducing vasoconstriction. T cells have been further implicated to mediate—at least partially- angiotensin II induced thrombosis through IL-6 by means of an adoptive transfer model into Rag1^−/−^ mice; however, this study did not directly implicate the AT1 receptor on the T cell, indicating a need for further mechanistic confirmation between IL-6 production and AT1 agonism on T cells (17). In the DOCA + salt mouse model of hypertension, the AT1 receptor has been demonstrated to polarize macrophages into the M1 phenotype following agonism with Angiotensin II through the HIF1α/ NF-κβ pathway resulting in vascular dysfunction and injury (162). Likewise, antagonizing AT1R reduced numbers of dendritic cells and reduced chemokine production in an experimental model of autoimmune encephalomyelitis, indicating this receptor can contribute to dendritic cell-mediated inflammation (163). Activated dendritic cell numbers increase in angiotensin-II induced hypertension (164), and Nahmod et al., found that DC differentiation is directly regulated by angiotensin II (165). Taken together, these findings indicate ARBs may elicit some of their antihypertensive effects through direct immune-modulation in addition to blocking the vasoconstrictive effect of angiotensin II. Further studies are needed to clarify some of these alternative mechanisms of action; however, it appears reasonable to anticipate that some of the antihypertensive effects of ARBs may be mediated through non-traditional mechanisms.

### Diuretics

The presence of a bumetanide sensitive channel on mouse macrophages (J774.2) was identified as early as 1985 by Bourrit et al. (166) NKCC1, an isotype of NKCC2 (targeted by loop diuretics such as furosemide or bumetanide), has been found on macrophages; indeed, antagonism with bumetanide resulted in blunted macrophage LPS-induced activation in the RAW264.7 mouse macrophage cell line (167). The presence and functionality of a channel on macrophages targetable by bumetanide and resulting in attenuated activation following stimulation suggests a potential for loop diuretics to blunt macrophage involvement in the progression of hypertension.

ENaC, therapeutically targeted by mineralocorticoid receptors (MR) blockers such as spironolactone to reduce expression indirectly or direct blockers such as amiloride, has been suggested to be expressed on dendritic cells (39), macrophages (168), and neutrophils (upregulated in hypertension) (169), playing a crucial role in regulating their involvement in hypertension. On dendritic cells, an amiloride sensitive channel has been demonstrated to regulate NADPH oxidase production, influencing isolevuglandin-protein adduct formation, T cell recruitment, and activation (39). These protein adducts may play a critical role in driving T cell involvement in the pathogenesis of hypertension (37, 170); as such, systemic administration of amiloride may lower blood pressure at least partially by reducing dendritic cell-to-T cell recruitment in hypertension, but further experimentation is necessary for confirmation if such an off-target mechanism is clinically relevant. In macrophages, a similar role for amiloride sensitive channel promotion of the inflammatory response has been characterized as well, indicating that systemic administration of amiloride may reduce macrophage inflammatory responses which have been demonstrated to play a role in the progression of hypertension (36, 86, 89).

The mineralocorticoid receptor has been found to be expressed on cells of T cells, B cells (171), and myeloid lineage (172), contributing to macrophage-induced cardiac hypertrophy in the angiotensin II + L-NAME mouse model of hypertension (172). Deficiency of this receptor on myeloid cells resulted in reduced aortic thickness, vascular damage, and fibrosis despite similar blood pressure elevation to L-NAME + AngII, illustrating the MR receptor on macrophages may be contributing to some of the inflammation observed in the heart during hypertension (172). In like manner, antagonism of the MR receptor with Finerenone or eliminating myeloid mineralocorticoid receptor expression has been shown to reduce the pro-inflammatory macrophage population in mouse and Large White Pig models, protecting against AKI-induced chronic kidney dysfunction (90). This correlation between myeloid MR reduction in function and reduced macrophage inflammation suggests that current mineralocorticoid receptor antagonists may partially reduce blood pressure and tissue inflammation through alteration of macrophage function within the kidneys and heart. If such a non-classically predicted effect of antihypertensives can be verified, it would further highlight the inextricable connection between hypertension and inflammation within the kidneys and heart.

### Beta adrenergic receptor blocker

β2-adrenergic receptors, targeted by beta blockers such as propranolol leading to reduced cardiac output and vasodilation (173, 174), have been found on B cells, CD4^+^ T cells, Th1 cells, CD8^+^ T cells, and macrophages (1, 175). The role of catecholamines (such as norepinephrine or epinephrine) on these cells due to beta adrenergic stimulation varies based on cell type (1). In CD4^+^Ts, for example, norepinephrine (NE) may inhibit IFNγ production through the β2 receptor (176); however, human CD8^+^Ts (particularly memory CD8Ts) responded to NE with increased inflammatory cytokine production (177). T effector function has been modulated successfully through NE-induced reduction in dendritic cell IL12p70 secretion leading to reduced IFNγ and higher IL-17 production in T cells following TCR stimulation by the LPS and NE exposed DCs (178). Although many of the nuances of this regulatory relationship between β2AR signaling and cytokine production are not yet understood, it is clear that such a relationship exists (1) and may influence innate-to-adaptive immunity recruitment in pro-inflammatory situations (178). Further research is needed to identify the effect of β-blockers on influencing the immune interactions contributing to blood pressure elevation.

### L-type calcium channels blocker

L-type calcium channels (LTCCs), blocked by calcium channel blockers such as amlodipine resulting in slower depolarization of vascular smooth muscle cells and conduction in the atrioventricular node (173), have been found expressed on macrophages (179), T cells (180), dendritic cells (Cav1.3) (181), and B cells (182). A hallmark signal of activation within immune cells involves an influx of calcium within the cytosol—either from extracellular sources or from a combination of release from intracellular stores and extracellular-to-cytosolic influx (183–187). The presence of these functional calcium channels on immune cells and their potential contribution to calcium influx lends credibility to the possibility that inhibition of LTCCs may influence immune cell proliferation and activation (181, 188). Evidence has been provided clinically by -perhaps somewhat surprisingly- dentistry wherein CCBs result in increased risk of gingivitis in patients, something also associated with immunosuppression (189, 190). This excellent 2013 review by Badou et al., outlines several mechanisms in T cells in particular (180); however, further research is needed to elucidate the precise effects inhibition of LTCCs plays on immune function and if some of the antihypertensive effects of calcium channel blockers are arbitrated through alteration of immune function.

## Current immune-regulating antihypertensive therapeutic treatments studied and complications

Clinically, elevated immune cell activation/function of CD3^+^/CD4^+^ (191), CD3^+^,CD8^+^ (99), and monocytes (192) have been described in hypertension. Upregulation of the cytokines IL-17 and IFNγ (21), IL-6 and TNFα (44), and IL-18 (18), has been correlated with clinical manifestation of essential hypertension, among other cytokines (193). To this end, targeting the immune system to reduce cytokine production and immune cell activation and infiltration may reduce blood pressure and subsequent tissue damage. This excellent review by Murray et al., describes the effects of immunosuppressants on blood pressure in animal models (193); however, clinical trials have been complicated through the nephrotoxic (and other tissue) effects of several immunosuppressants -such as calcineurin inhibitors- resulting in blood pressure elevation driven by tissue damage and sodium transporter upregulation (194–196). Our lab has recently identified the critical role tubular PD-L1 plays in mediating CD8^+^T-distal convoluted tubular interaction and hypertension; by knocking down PD-L1 in mice using renal tubule specific nanoparticles (197, 198) we were able to blunt blood pressure elevation in the DOCA + salt or adoptive transfer models of hypertension, identifying another potential therapeutic target (10). Further studies are needed to address the potential effectiveness of alternative immunosuppressant therapies in reducing blood pressure. Several promising pre-clinical studies have been conducted indicating the feasibility of using FDA-approved nanodrug platforms to deliver tissue specific immunosuppressant therapy (minimizing off-target toxicities), but further validation and studies are required before use in a clinical setting (199). Regardless of current difficulties, targeted immunosuppression to reduce inflammation and immune-mediated organ dysfunction in hypertension continues to be of scientific interest.

## Summary

The immune system plays a role in the pathogenesis of hypertension through several inflammatory signaling mechanisms involving cells from both the innate and adaptive immune system; however, the signaling molecules and pathways governing these interactions have proven to be complex and not yet fully understood. Within both the heart (109) and kidney (200), current data suggests higher pressure can, alone, drive immune infiltration and subsequent inflammation within the invaded organ leading to dysfunction. Immunodeficient mice (Rag2^−/−^) exhibited reduced pressure-driven cardiac remodeling (109), and the immunosuppressant tacrolimus reduced T cell infiltration, renal damage, and blood pressure elevation in the Dahl salt-sensitive rat on high salt diet (95). The immune system not only contributes to blood pressure elevation (35) but also mediates organ dysfunction and dysregulation initiated by elevated blood pressure (94). This inextricable connection between immunity, hypertension, inflammation, and organ dysfunction lends high priority to targeting the immune system to lower blood pressure or, at least, reduce inflammation due to hypertension (47). Even current FDA approved anti-hypertensives may mediate some of their beneficial effects through immune modulation, but further studies are needed for confirmation (157). Targeting the immune system to lower blood pressure and reduce organ damage has proven complicated (201, 202), but organ specific immune targeting using nanotechnology appears to be a promising solution to reduce toxicities (155, 199). Recent identification of single nucleotide polymorphisms in SH2B adaptor protein 3 contributing to T cell involvement in hypertension and renal damage (203) further complicates therapeutic targeting as genetic mutations may predispose certain immune cells to promote inflammation, supporting consideration of individual patient genetic predispositions when identifying driving factors of hypertension to design future treatment plans, as is becoming increasingly debated (204). As contributing immune players and inflammation-mediating molecules are characterized, novel pathways can be identified and targeted therapeutically to lower blood pressure and attenuate hypertension-mediated organ dysfunction. Recent studies have highlighted several such immunity-associated relevant receptors or cytokines and confirmed their relevance in several animal models of hypertension.
